# Primary Orbital Melanoma: Presentation, Treatment, and Long-term Outcomes for 13 Patients

**DOI:** 10.3389/fonc.2017.00316

**Published:** 2017-12-18

**Authors:** Anna M. Rose, Philip J. Luthert, Channa N. Jayasena, David H. Verity, Geoffrey E. Rose

**Affiliations:** ^1^Orbital Service, Moorfields Eye Hospital, London, United Kingdom; ^2^UCL Institute of Ophthalmology, London, United Kingdom; ^3^Department of Medicine, Imperial College London, London, United Kingdom

**Keywords:** primary orbital melanoma, orbital malignancy, ocular melanoma, melanoma, orbital surgery

## Abstract

**Background:**

Periocular melanoma is a rare but often deadly malignancy that arises in the uvea (commonest origin), conjunctiva or orbit (rarest primary site). Melanoma accounts for 5–10% of metastatic/secondary orbital malignancies, but only a tiny proportion of primary orbital neoplasia. Primary orbital melanoma (POM) is exceedingly rare, with approximately 50 cases reported to date.

**Methods:**

All patients seen in the orbital unit at a tertiary referral hospital (1991–2016) with a biopsy-proven diagnosis of POM were identified from a diagnostic database and were studied. The case notes, imaging, surgical approach, and histology were reviewed.

**Results:**

Thirteen patients (five male; 38%) presented with isolated malignant melanoma of the orbit, for which no other primary site was identified at presentation or during an average follow-up of 44 months (median 22; range 0–13 years). The patients presented between the ages of 40 and 84 years (mean 55.5; median 48 years) and typically gave a short history of rapidly increasing proptosis and eyelid swelling. On the basis of history, a malignant lesion was suspected in most patients and all underwent incisional biopsy, with debulking of the mass in 10 (77%) patients, and skin-sparing exenteration in 3/13 (23%). Ten patients underwent orbital radiotherapy and the survival to date ranged from 9 months to 14 years (mean 55 months; median 23 months); two patients received solely palliative care for widespread disease and one patient refused orbital radiotherapy. Five of the 13 (38%) patients died from the disease.

**Discussion:**

POM is a very rare malignancy, but clinical analysis of this cohort gives insight into disease presentation and prognosis. The tumor typically presents with a rapidly progressive, well-defined mass that is, in some cases, amenable to macroscopically intact excision. Unusual for malignant melanoma, some of these patients can show an unusually long period of quiescent disease after surgical debulking and radiotherapy.

## Introduction

Periocular melanoma is a rare, generally lethal, malignancy that can arise from in the eye (uveal tract), the conjunctiva, or the ocular adnexa (eyelid or orbit) (1). Uveal origin—from the iris, ciliary body or choroid—is the commonest ocular melanoma, with conjunctival melanoma being the second most frequent. The reported incidence of choroidal melanomas has increased in recent decades, possibly because of greater exposure to UV light (2), or possibly due to greater detection. Eyelid melanomas are very rare, with knowledge limited to small case series (3–5).

Orbital melanoma occurs either as primary disease, as secondary disease (local invasion from a uveal, conjunctival, or eyelid primary tumor), or as metastatic disease from distant origins such as skin. Melanoma accounts for 5–20% of metastatic and secondary orbital malignancies, but only a minute proportion of primary orbital neoplasia (6–11). Primary orbital melanoma (POM) is extremely rare, with only about 50 cases reported to date (Table 1), and is thought to arise from melanocytic cells of the leptomeninges or ciliary nerves, or from ectopic intraorbital nests of melanocytes (12). POM can occur *de novo*, but it is often reported in association with pigmentary changes within periocular tissues—such as nevus of Ota, blue cellular nevus, or oculo-dermal melanosis; indeed, over a half of patients with nevus of Ota have pigmentation within the orbit, including oculo-dermal melanosis (13). While POM is reported to have a very poor prognosis, there have been sporadic reports of long-survival, such as one patient who lived for almost 30 years after initial diagnosis (14).

**Table 1:** Summary of published cases of primary melanoma within the orbit.

Number of cases	Gender	Age of onset	Side	Other features	Reference
1	M	34	L		(15)
1	F	8	R	Giant divided nevus	(16)
1	F	34	L		(17)
1	M	45	L	Nevus of Ota	(18)
1	M	60	L	Poliosis	(19)
1	M	50	R		(20)
1	M	22	R		(21)
1	F	64	R		(22)
1	M	59	L		(23)
1	F	43	R	Blue nevus	(24)
10	–	Mean age = 57	–	Survey of >1,200 orbital neoplasia	(25)
1	F	36	R	Episcleral nevus	(26)
1	M	40	L		(27)
3	F	45	Unknown	Orbital nevus	(28, 29)
	F	33	Unknown	Orbital nevus	
	M	43	R	Orbital nevus	
1	M	29	L	Ocular melanosis	(30)
1	F	49	L	Ocular melanosis	(31)
1	F	5	L		(32)
21	–	Mean age = 42	–	Review of national pathology registry showed 19/21 had blue nevus	(33)
1	M	79	L		(30)
1	M	76	R		(34)
2	M	46	Unknown		(35)
	M	59	Unknown		
1	F	17	R	Ocular melanosis	(36)
1	M	27	R	Blue nevus	(37)
55 cases	(14 M, 10 F)	Mean = 44.4 years	(10R, 10L)	–	–

Based on a series of patients with POM, this work aimed to extend the knowledge about clinical presentation, radiological appearance, surgical approach, and prognosis for this extremely rare condition.

## Patients and Methods

All patients with a biopsy-proven diagnosis of POM, seen for diagnosis and treatment at Moorfields Eye Hospital between 1991 and 2016, were identified from a clinical orbital diagnostic database and included in the study. The database comprised all patients seen by the orbital service at Moorfield’s Eye Hospital with a diagnosis of orbital malignancy, both primary and secondary. The histology slides were reviewed by an orbital malignancy-expert pathologist, and where diagnostic uncertainty existed, a second opinion was sought by a melanoma-expert pathologist. The radiological imaging and clinical case notes were reviewed. Metastasis from a distant site was excluded by a thorough clinical, ultrasonographic, and (where available) histological examination of both uveal tracts, with a complete skin survey by consultant dermatologists at the patient’s local hospital, by review of systemic health during the follow-up interval, and by CT, MR, and, in some cases, PET imaging. This study received ethics approval from Moorfields Eye Hospital Biobank ethics board (15/SW/0104).

## Results

Thirteen patients (five men; 38%) with POM were identified, their mean age at presentation being 55.5 years (median 48; range 40–84 years) (Table 2). Twelve of the patients were white European, while one patient was West African (Table 2). The left orbit was more commonly affected (eight cases; 62%), and three patients had an underlying localized pigmentary abnormality (one nevus of Ota, one conjunctival nevus, one oculo-dermal melanosis).

**Table 2:** Clinical characteristics of 13 patients with primary orbital melanoma.

Case no.	Gender	Age at onset (years)	Side	Primary treatment of orbital disease	Orbital progression	Time orbital treatment to orbital recurrence (months)	Systemic disease at presentation	Systemic progression	Time orbit to systemic disease (months)	Systemic therapy	Survival from orbital onset (months)	Age at death (years)	Notes
1	F	81	R	Debulking	N	–	Liver, regional lymph nodes	Y	0		3	81	Too unwell for adjuvant radiotherapy
2	M	40	L	Exenteration	N	–	Liver, regional lymph nodes	Y	0		4	40	Too unwell for adjuvant radiotherapy
3	F	48	L	Debulking + RT	N	–		N	–		19	Alive	
4	F	58	L	Exenteration	Unknown	–		Unknown	–		22	Alive	Patient declined adjuvant radiotherapy
5	M	45	L	Debulking + RT	Y	6		N	–		24	Alive	Exenteration after orbital progression
6	F	84	R	Exenteration + RT	N	–		Y	12	Palliative RT for bone metastases	25	85	Conjunctival melanosis
7	F	60	R	Debulking + RT	Y	–	Temporal lobe	Y	0	Nil active	22	63	
8	M	45	L	Debulking + RT	Y	7		Y	45	Liver resection	78	Alive	Nevus of Ota Exenteration after orbital progression
9	F	47	L	Debulking + RT	Y	161		Y	168	Nil active	174	60	Conjunctival nevus Late exenteration
10	M	46	R	Debulking + RT	N	–		N	–		13	Alive	
11	F	43	L	Debulking + RT	N	–		N	–		175	Alive	West African
12	F	70	L	Debulking + RT	N	–		N	–		9	Alive	
13	M	55	R	Debulking + RT	Y	2		Y	5	Immunotherapy	12	Alive	Immunotherapy for progressive systemic and orbital disease

*“RT” denotes fractionated external beam radiotherapy. All patients detailed in this table have given their written informed consent for the publication of this data*.

### Clinical Presentation and Imaging

**Figure 1:**
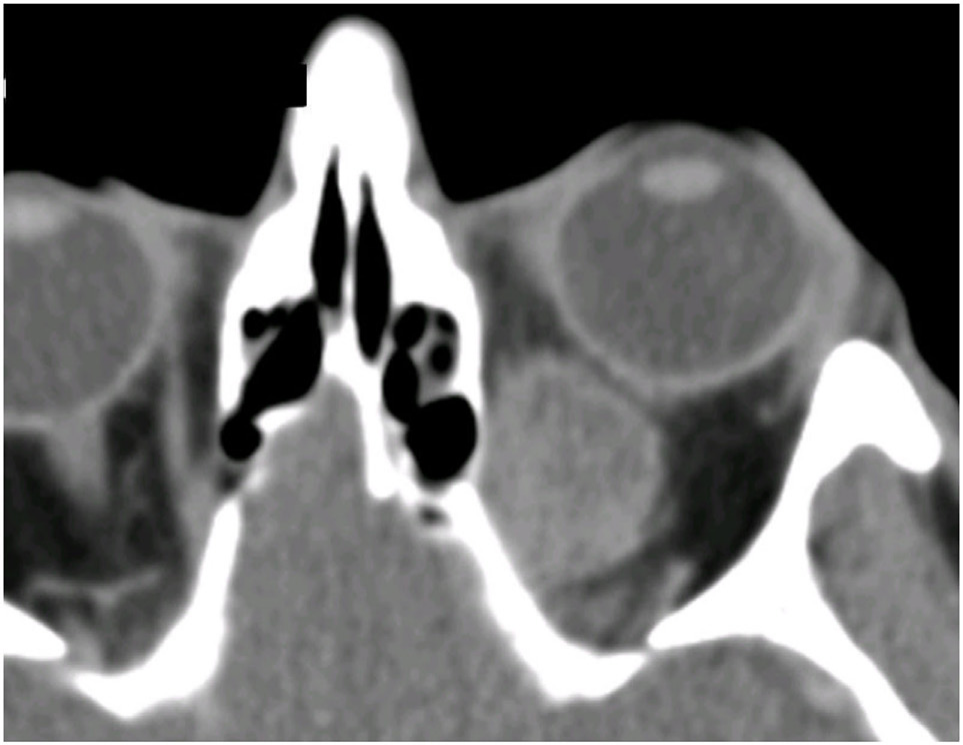
CT scan showing left orbital melanoma, with a typical configuration suggesting a well-defined benign mass; the rapid onset of symptoms over a few months, however, belies the sinister nature of the condition.

All 13 patients presented with a history of unilateral proptosis with varying degrees of diplopia, and retro-orbital, or periorbital pain. The proptosis was generally rapidly progressive (over 2–6 months), but in two cases, it had progressed slowly over 4–5 years (patients 4 and 11; Table 2). Imaging consistently showed a relatively well-circumscribed, enhancing soft-tissue lesion that resembled benign lesions such as cavernous hemangioma (Figure 1).

### Treatment

**Figure 2:**
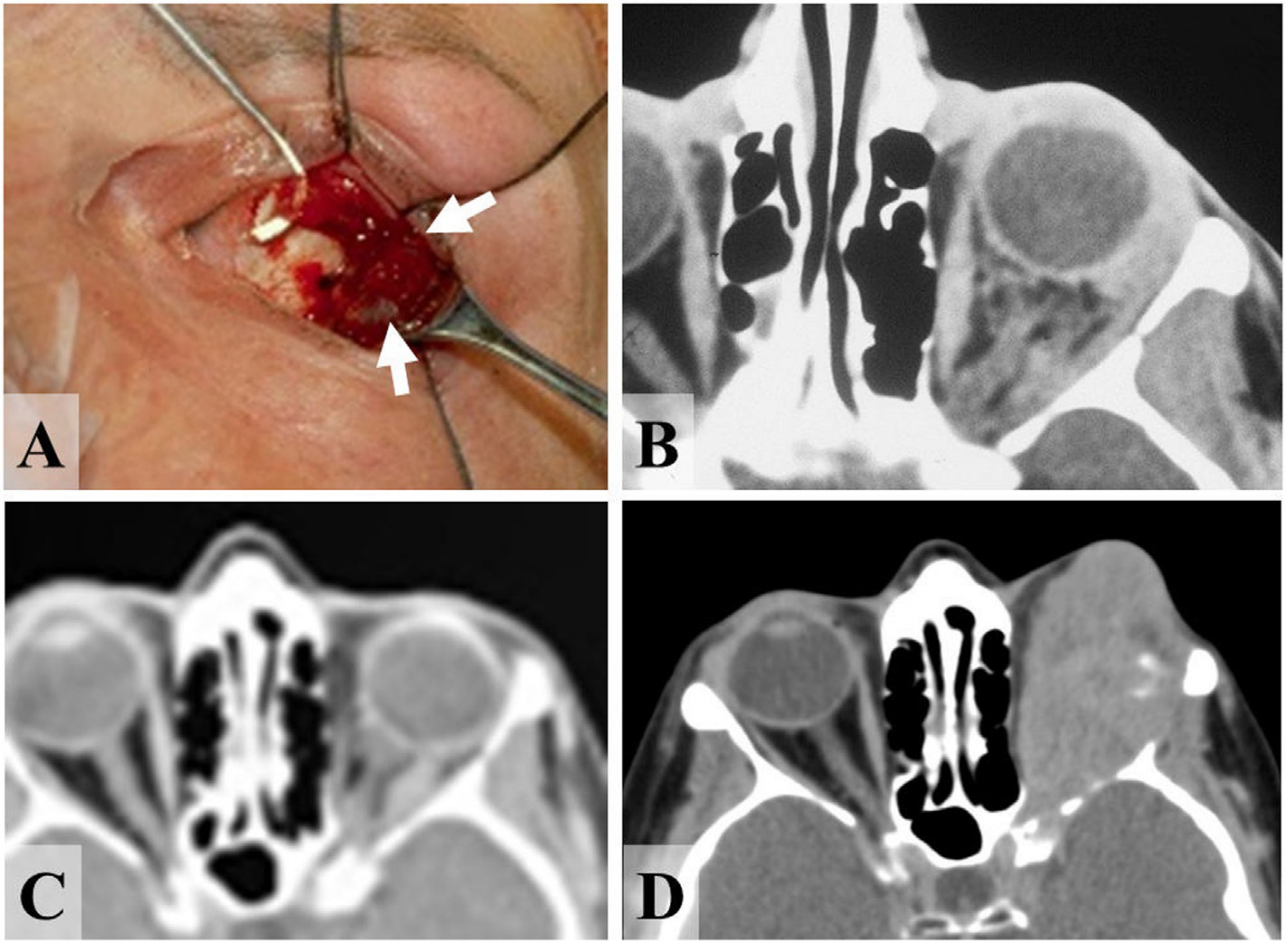
47-year-old female presenting with primary malignant melanoma of the left orbit. **(A)** Melanoma (arrows) throughout orbital fat alongside the lateral rectus (on squint-hook) at time of incisional biopsy, and **(B)** CT scan of orbits prior to diagnostic biopsy, showing diffuse tumor infiltration of the left retrobulbar fat. **(C)** CT at 8 years after orbital radiotherapy, showing the inactive orbital disease that persisted for more than 13 years. **(D)** After 13 years there was a very rapid recurrence of tumor, from which the patient died about a year later.

Diagnostic anterior orbitotomy and biopsy was done in all cases and a highly pigmented tumor was evident at surgery in ten cases (Figure 2A), and the other 3 patients (nos. 1, 7, and 13; Table 2) had lightly colored purple lesions. Well-defined lesions underwent macroscopically intact excision, whereas diffuse or infiltrative lesions had all visible tumor removed piecemeal (“debulked”). Primary orbital exenteration was *not* routinely performed for three reasons: first, primary debulking or intact excision effectively addresses the disease focus at initial surgery; secondly, there is no current evidence that exenteration—necessitating a second procedure—improves patient survival or reduces local disease recurrence; thirdly, our use of anterior orbitotomy (without disruption of bone or periosteum) still permits future orbital exenteration if required for local progression of disease. Skin-sparing exenteration was performed in three cases and a fourth patient (Patient 4) underwent exenteration 6 months after initial debulking, due to progression of the orbital disease. All patients were considered for adjuvant radiotherapy, but two patients (Cases 1 and 2) were too unwell to receive treatment, and Patient 4 declined it; the remaining nine patients received orbital radiotherapy at about 2–3 months after surgery, the standard protocol being 50–55 Gy in 200 cGy fractions over 5 weeks. Of the 13 patients, nine individuals received primary treatment in the period before monoclonal antibody therapies became available for malignant melanoma. The four patients treated in recent years were genetically typed for likely response to monoclonal antibody therapy by standard methods in a diagnostic lab, and it was found that only one patient (patient 13) was likely to benefit.

### Clinical Course and Outcome

Eight of the thirteen patients are currently alive (at time of manuscript submission), with a mean survival of 44 months (median 22; range 9–175 months) and five have died from the disease—with highly variable survival between 3 and 174 months after diagnosis (mean 44; median 16 months) (Table 2).

Three patients had systemic involvement at presentation: Patients 1 and 2 had hepatic metastases and regional lymph node involvement, and both died within 4 months; patient 7 had temporal lobe metastases at presentation and died 22 months later. Patient 8 developed a liver metastasis at 45 months, underwent partial hepatectomy, and remains in remission at 77 months after the primary orbital presentation. Patient 13 developed liver metastases at 5 months after orbital surgery and is currently being treated with monoclonal antibody therapy, with significant tumor regression.

Three patients had progressive or recurrent orbital disease, this being about 6 months after orbital diagnosis in two patients (Cases 5 and 8; Table 2). The third patient (Case 9) is known to have had diffuse and widespread orbital infiltration at primary surgery (Figures 2A,B), but remained with clinically and radiologically inactive orbital disease for 161 months (Figure 2C) before developing a rapidly progressive local recurrence that required multiple orbital tumor resections (Figure 2D); after inactive tumor for 13 years, the patient died with from disseminated malignancy within 13 months of orbital reactivation.

The eight currently alive patients have a wide variation in follow-up times (9–72 months), but the side-effect profile of debulking surgery with adjuvant radiotherapy appears to be good. There have been no complaints of serious post-operative pain or diplopia. Visual acuity has been affected in all individuals, but to varying extents, but some useful vision was retained in all patients after debulking surgery and radiotherapy.

## Discussion

We present the clinical characteristics, treatment approaches, and long-term outcomes for 13 patients with POM, this representing the largest clinical series for this disease. Previous solitary case reports are consistent with our demographic findings—namely that most individuals are of white Northern European descent and present from the 5th decade (median age 48 years; range 40–84). One patient was West African and this would appear to be unique. Review of previously published cases suggests onset at a mean age of 44 (median 42 years), with the youngest case occurring in an 8-year-old girl and the oldest patient being 79 years (Table 1) (16, 38). Interestingly, two of our patients presented in their ninth decade, this being exceptionally late for POM. There does not appear to be any gender bias—with 19 affected men (14 previously reported + 5 in this study) and 18 affected women (10 previously reported + 8 in this study).

Imaging of patients consistently showed a well-circumscribed lesion that looked typically like a benign tumor or arteriovenous malformation; this characteristic has been previously described and might lead to delay in diagnosis and treatment of disease (39). MRI signal characteristics will generally help differentiate melanoma from benign lesions, such as cavernous hemangioma and could be considered in cases where there is diagnostic uncertainty (39). Our patients all had incisional biopsy and, where possible, resection of the mass, and all were considered for high-dose fractionated orbital radiotherapy to attempt control of residual local disease. Six patients underwent exenteration—three (Cases 2, 4, and 6) to control disfiguring orbital disease, two (Cases 5 and 8) for rapid disease progression after initial debulking, and one patient (Case 9) for very late recurrence of the orbital disease.

Despite reasonably uniform management, the outcome for this cohort of POM patients was highly variable: for example, two patients died very shortly after diagnosis, while three have survived 6 years or more. It would seem that this variable course does not depend solely on the presence of metastasis or systemic progression, as one of the longest surviving patients (Case 8) had partial hepatectomy for a liver metastasis. The surviving group (eight individuals, with three of these progressing to exenteration) was insufficiently large to draw conclusions about long-term visual outcomes in this patient group. There have, however, been other larger studies of more common orbital malignancies treated with a combination of surgery and radiotherapy that have shown good visual outcomes. For example, in a study of orbital rhabdomyosarcoma, one third of patients maintained vision better than 6/9 in their treated eye, and approximately half maintaining vision of 6/9–6/60 (40).

Choroidal melanomas have been broadly classified as “type I” or “type II,” these following “aggressive” or “relatively indolent” courses, respectively. Several genetic signatures have been found for “type I” tumors—with monosomy 3, present in a half of uveal melanomas, being the most significant chromosomal aberration and strongly associated with metastasis and death (41, 42). Monosomy 3 affects prognosis due to tumor haploinsufficiency of *BAP1*—an important *BRCA1*-associated tumor suppressor gene (43, 44). Other chromosomal abnormalities, such as loss of 6q and gain of 8q, have been associated with poor prognosis in uveal melanoma (41). Point mutations in *GNAQ* and *GNA11* have been identified in 80–90% of uveal melanomas and lead to activation of the MAPK/MEK/ERK pathway (43). It would be valuable to establish whether these genetic aberrations are present in POMs, and whether these relate to prognosis.

One patient (Case 9) is particularly interesting: despite receiving only orbital radiotherapy for widely infiltrating melanoma at the time of diagnosis (Figures 2A,B), she remained with no evidence of tumor proliferation until more than 13 years later. After tumor reactivation, however, it followed a very aggressive course (Figure 2C), requiring several palliative procedures, and the patient died 13 months later. Late metastatic or secondary melanoma to the orbit has been reported (45, 46), and similar genetic factors might possibly control the late recurrence of both primary and secondary orbital melanomas; alternatively, the orbital milieu might pre-dispose to a prolonged tumor latency before late recurrence.

In summary, POM is an extremely rare malignancy of rather variable prognosis after treatment, for which local resection with adjuvant radiotherapy remains the mainstay of therapy. Disease can remain quiescent for extended periods of time before following an aggressive course. Immunotherapy might play a role in the future, but in this cohort, genetic testing did not suggest response to currently available agents in the majority of individuals. Further genetic investigation of these rare tumors might elucidate underlying molecular mechanisms of oncogenesis, thereby improving prognostication and treatment for this patient group.

## Ethics Statement

This study received ethics approval from Moorfields Eye Hospital Biobank ethics board (15/SW/0104).

## Author Contributions

Concept and design of study—AR, CJ, DV, and GR. Clinical/histological examination of patients—PL, DV, and GR. Data collection—AR. Data analysis—AR and GR. Manuscript preparation and review—AR, PL, CJ, DV, and GR.

## Conflict of Interest Statement

The authors declare that the research was conducted in the absence of any commercial or financial relationships that could be construed as a potential conflict of interest.
